# Thermal Cameras for Continuous and Contactless Respiration Monitoring

**DOI:** 10.3390/s24248118

**Published:** 2024-12-19

**Authors:** Raquel Alves, Fokke van Meulen, Sebastiaan Overeem, Svitlana Zinger, Sander Stuijk

**Affiliations:** 1 Department of Electrical Engineering, Eindhoven University of Technology, 5600 MB Eindhoven, The Netherlands; 2 Centre for Sleep Medicine Kempenhaeghe, 5590 AB Heeze, The Netherlands

**Keywords:** thermal cameras, respiration rate, remote thermography, respiratory flow, respiration monitoring

## Abstract

Continuous respiration monitoring is an important tool in assessing the patient’s health and diagnosing pulmonary, cardiovascular, and sleep-related breathing disorders. Various techniques and devices, both contact and contactless, can be used to monitor respiration. Each of these techniques can provide different types of information with varying accuracy. Thermal cameras have become a focal point in research due to their contactless nature, affordability, and the type of data they provide, i.e., information on respiration motion and respiration flow. Several studies have demonstrated the feasibility of this technology and developed robust algorithms to extract important information from thermal camera videos. This paper describes the current state-of-the-art in respiration monitoring using thermal cameras, dividing the system into acquiring data, defining and tracking the region of interest, and extracting the breathing signal and respiration rate. The approaches taken to address the various challenges, the limitations of these methods, and possible applications are discussed.

## 1. Introduction

Monitoring respiration has a fundamental role in healthcare, providing valuable insights into an individual’s physiological state. Respiratory rate, volume, and pattern can be crucial indicators of the person’s health status, early warning signs, and potential diagnosis of underlying medical conditions. For example, monitoring respiration is essential in critical care settings, where changes in breathing patterns may signal impending respiratory distress or failure. Another example is in distress situations of patients suffering from chronic diseases like chronic obstructive pulmonary disease (COPD) or asthma, where continuous respiratory monitoring helps in the management of the diseases, allowing timely intervention and improving patient outcomes. Similarly, premature babies in the neonatal intensive care unit (NICU) require continuous monitoring of respiration to prevent scenarios of hypoxia or apnea of prematurity [1]. Furthermore, in the case of diagnosing breathing-related sleep disorders, monitoring respiration during sleep is essential to establish a diagnosis and evaluate the treatment process [2,3,4,5].

There are different techniques to continuously monitor respiration that vary in their invasiveness, precision, applicability to different clinical situations, and cost of implementation. The choice of monitoring method and the gold standard methods depend on the specific needs of the patient, the clinical setting, and the information required by healthcare providers. Furthermore, when talking about respiration monitoring, various quantities could be measured here. This includes respiration rate (number of breaths per minute) as well as other quantities describing the respiratory quality, like respiratory effort, volume of air inhaled or exhaled, percentage of gases or saturated hemoglobin in the blood, ${\mathrm{CO}}_{2}$ saturation during exhalation, and lung volumes and capacities [5].

The method considered as the gold standard to monitor respiration continuously is the end-tidal ${\mathrm{CO}}_{2}$ technique. The end-tidal ${\mathrm{CO}}_{2}$ technique (${\mathrm{EtCO}}_{2}$) is performed through endotracheal intubation or through capnography where it measures the partial pressure of exhaled ${\mathrm{CO}}_{2}$ [6]. As the technique might be rather obtrusive for continuously monitoring respiration, it is primarily used to monitor patients when endotracheal intubation takes place [7].

A frequently used method for the evaluation of respiration quality in clinical practice is the use of pulse oximetry. This technique uses LEDs and photodiodes to measure the peripheral saturation of oxygen (${\mathrm{SpO}}_{2}$) by analyzing the light absorption ratio between the hemoglobin’s wavelength and deoxyhemoglobin’s wavelength [8,9,10]. This technique is often used to estimate other quantities (e.g., heart rate) and derived quantities (respiration rate) as well. The devices for pulse oximetry require skin contact with fingers, forehead, nose, foot, ears, or toes, and measurements can be affected by skin pigmentation, obesity, and hypotension [11]. These methods are contact-based methods that may restrict the patient’s movement, cause discomfort and skin irritation, and disturb the patient. For this reason, non-contact alternatives have been a topic of research since the late 1990s [12,13].

**Figure 1:**
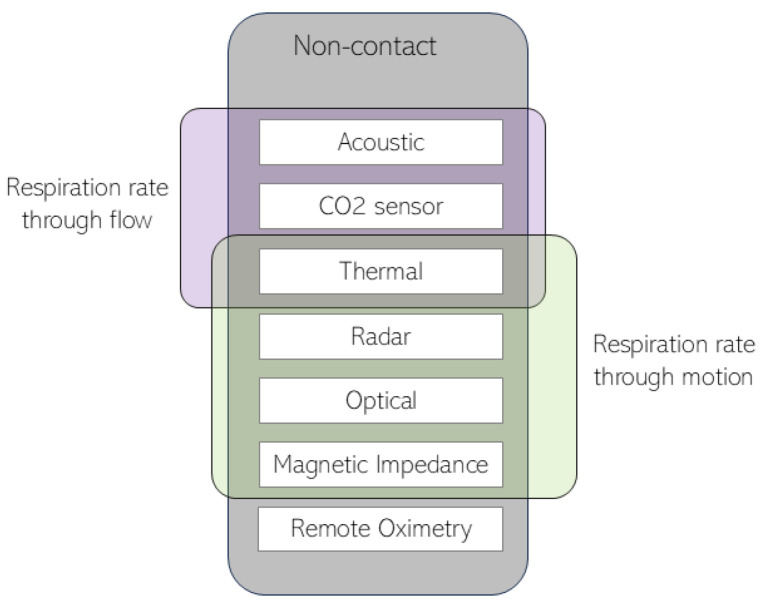
Non-contact respiration monitoring techniques divided into the respiration rate obtained through flow data and respiration rate obtained through motion data.

Most non-contact methods measure respiration rate either through the analysis of flow variations and/or the analysis of motion variation. Figure 1 shows how the different non-contact methods fit in this division. The respiration flow consists of the air that is inhaled and exhaled in each respiration cycle, whereas the respiration motion refers to the movement in the chest area during this cycle.

Acoustic sensors can be used to monitor respiration rate without direct contact, taking advantage of the breath-related acoustic signals caused by exhaled air turbulence. In [14], a speaker is used to transmit ultrasonic frequencies and microphones are used to detect reflections. In [15], the respiration rate is measured using a smartphone’s microphone placed 2 cm from the mouth/nose area. Nevertheless, these systems are extremely vulnerable to environmental noise, wind or airflow, patient movements, and microphone placement.

A promising technique to monitor respiration rate without contact is by measuring the ${\mathrm{CO}}_{2}$ concentration. This can be done either through a sensor placed between 20 and 60 cm from the mouth and nose [16] or through the use of thermal cameras operating in the ${\mathrm{CO}}_{2}$ absorption band [17]. Both approaches are currently in the development stage, but their practical use is conditioned by the considerable expense associated with the sensors, making them less attractive for clinical applications.

The second category in Figure 1 monitors respiration rate through motion. For example, respiration is measured through the use of radar technology. Radar detects respiration by analyzing reflected signals to estimate or evaluate chest movements during breathing [18,19]. Different radar types, like continuous-wave, frequency-modulated continuous wave, ultra-wideband, and Doppler radar, can be employed, and ongoing research aims to improve accuracy for non-invasive respiration monitoring [20].

Optical methods offer another option to quantify body movements caused by respiration, in a contactless manner. These methods, which are under development, include the use of video photoplethysmography [21,22] as well as fiber-grating vision sensors. These sensors change their characteristic wavelength response when exposed to external stimuli such as strain or temperature [23]. However, the downsides of these methods include their accuracy dependence on environment lighting and temperature conditions, as well as significant implementation costs [12,21].

Another method to monitor respiration consists of the use of magnetic impedance. This involves generating a magnetic field using coils and then measuring the resulting impedance changes that occur due to the movement of air in and out of the lungs. The sensors used for this technology can be placed in a bed’s mattress or a chair’s backrest [24,25,26], making this a promising way to monitor respiration unobtrusively.

An alternative variation of the optical methods is remote oximetry, which operates on the same principle as contact-based pulse oximetry. This technology compares light absorption at two wavelengths that correlate with the concentrations of oxygenated and deoxygenated hemoglobin. Variations in light absorption at these wavelengths generate a photoplethysmography (PPG) signal, which can be used to derive the respiration signal and respiration rate. Additionally, the ratio of these signals enables the calculation of ${\mathrm{SpO}}_{2}$ values [27,28].

The wavelengths used are usually within the visible and the near-infrared (NIR) spectrum, but recent studies have been trying to use remote oximetry using two different channels of RGB cameras [29,30,31]. Nevertheless, despite being still in development, this method is very sensitive to illumination (whether visible or NIR) and can even require an illumination source, restricting its application [30].

Finally, respiration can also be continuously monitored considering the variations of temperature between the inhaled and exhaled air. Thermal sensors, such as thermal cameras, measure the infrared radiation and correlate it to the absolute or relative temperature. This allows the measurement of temperature variations that can be associated with respiration movements, through the contrast between the body and the background conditions or clothing, or respiration flow through the differences of temperatures in the nostrils and mouth area [5]. Therefore, thermal cameras appear in an advantageous position since they use both the respiration flow data and the respiration motion data. This characteristic of thermography for respiration monitoring is particularly interesting for applications where the distinction between respiration motion and flow is important, for instance, in the distinction between different types of sleep apneas. In addition, this technology is not affected by lighting conditions or by the environmental temperature, within limits, unveiling its potential, especially for monitoring respiration during sleep.

The ultimate goal when monitoring respiration is to have a method that offers the advantages of both continuous and remote monitoring, enabling healthcare providers to accurately track respiratory parameters over extended periods, both in clinical settings and at home. Taking into account all of this, thermal cameras appear as a highly promising method. This work describes the steps and techniques applied for respiration rate monitoring with thermal cameras for clinical purposes. Considering that this is a field under development without large clinical trials or standard protocols, this overview summarizes the most relevant studies done up to this moment and gives insights into different applications and limitations. The focus of this overview is limited to studies that use thermal cameras alone instead of hybrid models so that the different challenges within each processing step can be properly understood and addressed.

## 2. Remote Thermography

**Figure 2:**
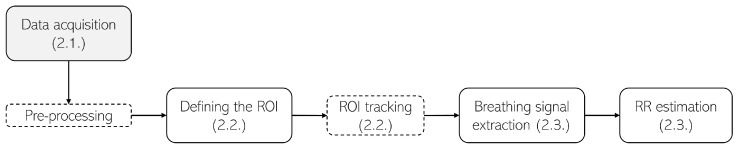
Pipeline for respiration rate monitoring and apnea detection in acquisitions with thermal cameras. Each step in the pipeline is described in the section indicated in parentheses.

Thermal cameras measure infrared radiation and correlate it to the absolute or relative temperature. The system for respiration rate (RR) estimation with thermal cameras can be divided into different stages, as shown in Figure 2. The initial step comprises the process of acquiring thermal image sequences, i.e., videos, with one or multiple thermal cameras. These can have different placements and can be facing the subject from different angles. In some cases, the thermal cameras may be used in parallel with other cameras, for instance, RGB cameras [32,33,34] or NIR cameras [35]. Depending on the cameras’ specifications and placement, the field of view may incorporate non-relevant elements and noise inducers to the breathing signal. Therefore, it is necessary to define a region of interest (ROI) where the pixels with potential breathing information are present. The approach used for monitoring can imply the need for an ROI tracking mechanism throughout the acquisition period. By evaluating the pixels’ intensity variation over time, within the ROI, the breathing signal can be estimated. This signal requires some filtering for noise reduction and pattern enhancement. The RR is calculated as the frequency of the breathing signal.

The general pipeline described above can be approached in several ways. Different studies have been done in order to find the best methods that value simplicity and/or accuracy. Table 1, Table 2 and Table 3 comprise different studies done, values measured, the used datasets, reference methods, as well as techniques used. The following sections describe how each study, chronologically organized, developed each of the pipeline’s steps.

### 2.1. Data Acquisition

Thermal cameras used for respiration rate monitoring are available in different price ranges, resolution ranges, and different specifications. Thermal cameras can measure either medium-wave infrared (MWIR) or long-wave infrared (LWIR) signals. MWIR cameras usually have a greater resolution than LWIR cameras. High-resolution cameras are costly and have as high as 1280 × 1024 pixels and reach frame rates of 30 Hz. As a low-cost alternative, low-resolution thermal cameras usually have resolutions of 160 × 120 pixels but can go as low as 60 × 80, and their frame rate is usually around 10 Hz. Low-resolution cameras pose a challenge regarding the proximity of the subject or the need for optical lenses to provide reliable measurements. There are also high-speed thermal cameras with frame rates above 100 Hz and with a resolution of around 512 × 640 pixels. However, for respiration monitoring, which consists of signals around 0.2 Hz, an increase in temporal resolution is not needed. So far, no researchers used cameras with these properties for respiration monitoring. For respiration monitoring, the thermal cameras used have a sensitivity between 0.025 °C and 0.070 °C. To convert a pixel intensity into a temperature value, there needs to be a calibration by setting an emissivity value or by using a reference object with known emissivity. However, in this application, the goal is to monitor temperature changes; therefore, only temperature differences are used. This is particularly important since, this way, there is no need to calibrate the thermal sensors and no need to set a value of emissivity. However, any disturbances during the recordings might affect the emissivity and therefore create momentarily inaccuracies.

Since 2000, Pavlidis and his group started applying the use of remote thermography to monitor vital signs [36,37,38]. In 2006, they began applying this technology to the measure of respiration. Murthy and Pavlidis et al. experimented with MWIR cameras (Phoenix camera [39] and FLIR SC-6000 [40]), both with 640 × 512 pixels. In this first study, the average breathing rate of three healthy participants was estimated. The camera was placed sideways to the patient at around 2 m distance at a specific viewing. As a reference method, a respiration belt sensor was used [39]. The average accuracy for this method was around 96%; nevertheless, the sample size was not significant and the conditions of the recording, specifically the camera placement and viewing angle, were very restrictive. In their follow-up study [40], the goal was to detect airflow abnormalities. For this, 14 healthy participants and 13 patients with obstructive sleep apnea (OSA) were recruited, and the measurement results were compared with the simultaneously collected polysomnography (PSG). They achieved a chance-corrected agreement (kappa) of 0.92 with the thermistor signal, 0.83 with the nasal pressure signal, and 0.80 with the expired ${\mathrm{CO}}_{2}$. Even though this was the first study that demonstrated feasibility in detecting airflow abnormalities with thermal imaging, the sample size is still low, the ground truth scoring was limited to apneas (pause in breathing) and hypopneas (shallow breathing or low breathing rate), and the classification was done manually.

Also in 2009, Fei and Pavlidis [41] used, as well, the FLIR SC-6000 MWIR camera with an indium antimonide (InSb) detector to measure the breathing rate of 20 healthy participants and compared the results with waveforms extracted from thermistors. The complement of the absolute normalized difference (CAND) obtained was 98.27%. However, the measurements were solely based on the nostrils, and the patients were asked to stay very still, which did not represent a daily life clinical setting.

In 2010, Al-Khalidi et al. [42] focused their study on detecting and tracking the region of interest (ROI) for respiration rate monitoring using the FLIR A40 thermal camera at 50 fps. The maximum failure rate obtained was 8%, presenting a reliable starting point for breathing monitoring.

Abbas et al. [43], in 2011, used the VarioCAM^®^, an LWIR camera, for the first time, measure the breathing rate of seven infants in the neonatal intensive care unit (NICU). Although it had a relatively small population size, this study showed the potential of neonatal monitoring with thermal cameras.

Lewis et al. [44] performed a study comparing two different thermal cameras when estimating the breathing rate and the relative tidal volume. The cameras used were the TVS-700 with twelve patients and the FLIR SC-6000 with seven patients. The ground truth was established through the LifeShirt^®^ system (Vivometrics, Inc., Ventura, CA, USA). For the TVS-700 camera, the mean correlation obtained for the breath interval was 0.98 and for the relative tidal volume was 0.90. For the SC-6000 camera, the mean correlation obtained for the breath interval was 0.95 and for the relative tidal volume was also 0.90. This study presented an interesting approach for tidal volume measurement; however, it used a reduced sample size and a specific environment.

Also in 2011, Goldman et al. [45] tested on 17 children with respiratory pathologies to measure respiration airflow and synchronicity of thoracoabdominal motion, since abnormal breathing can be associated with asynchronous movements of the thorax and abdomen. They used nasal pressure transducers and manual annotations as a reference. The results obtained show a Cronbach’s Alpha value of 0.976 between the measured signals and the references. In addition, they showed that the time delay observed between the ribcage and the abdomen in children with respiratory disease was significantly different from that of healthy children.

Considering a different type of application, Chauvin et al. [46] measured the RR of subjects biking on a stationary bike for telerehabilitation purposes. They tested 15 healthy participants with different facial characteristics and in 4 different conditions, using a respiration belt as a reference. The thermal camera was positioned in front of the bike in a pan–tilt unit. Chauvin et al. showed that monitoring the RR becomes a challenge once the subjects have glasses or facial hair. They obtained a wide range of *tpi*, the percentage of cycles where the error is lower than 1 bpm, within the different conditions. This proves the feasibility of using such technique in a high dynamic setting but highlights the different challenges that come inherent to it.

**Table 1:** Overview of the studies that include thermal cameras for the measurement of respiration rate and the detection of apnea events.

	Participants	Environment	Camera(s) (Type, Resolution)	Reference	Outcome	Flow/Motion Separation	Performance
Murthy et al. (2005/2006) [39,47]	3 healthy adults	Lab	MWIR, (640 × 512)	Respiration belt	RR	No	accuracy = 96.43%
Murthy et al. (2009) [40]	14 healthy adults 13 adults with OSA	Lab	MWIR, (640 × 512)	Polysomnography	Airflow abnormalities	No	kappa = 0.80–0.92%
Fei and Pavlidis et al. (2010) [41]	20 healthy adults	Lab	MWIR, (640 × 512)	Respiration belt	RR	No	CAND = 98.27%
Al-Khalidi et al. (2010) [42]	-	Lab	LWIR, (320 × 240)	-	ROI for RR	No	-
Abbas et al. (2011) [43]	7 infants	NICU	LWIR, (1024 × 768)	Chest impedance ECG monitor	RR	No	-
Lewis et al. (2011) [44]	12 healthy adults 7 healthy adults	Lab	LWIR, (320 × 240) MWIR, (640 × 512)	Plethysmography	RR, IBI, rTV	No	correlation = 0.90–0.98 correlation = 0.90–0.95
Goldman et al. (2012) [45]	17 children	PEDS	LWIR, (320 × 240)	Nasal pressure Manual annotations	RR, breathing synchronicity	Yes	alpha = 0.976
Chauvin et al. (2014) [46]	15 healthy adults	Lab	LWIR, (640 × 480)	Respiration belt	RR	No	*tp2* = 37–100%
Pereira et al. (2015) [48]	11 healthy adults	Lab	LWIR, (1024 × 768)	Piezo-plethysmography	RR/IBI	No	correlation = 0.940–0.974 MAE = 0.33–0.96 bpm
Ruminski et al. (2016) [49,50]	16 healthy adults 12 healthy adults	Lab	LWIR, (320 × 240)	Respiration belt	RR, apneas	No	MAE = 0.415–1.291 bpm correlation = 0.912–0.953%
Pereira et al. (2017) [51]	12 healthy adults	Lab	LWIR, (1024 × 768)	Piezo-plethysmography	RR	Yes	correlation = 0.95–0.98 RMSE = 0.28–3.45 bpm
Ruminski et al. (2017) [52]	10 healthy adults	Lab	LWIR, (60 × 80)	Respiration belt	RR	No	MAE = 0.236–0.350 bpm
Pereira et al. (2018) [53]	20 healthy adults	Lab	MWIR, (1024 × 768)	Piezo-plethysmography	RR (and HR)	No	RMSE = 0.71 ± 0.30 bpm
Pereira et al. (2018) [54]	12 healthy adults 9 newborns	Lab NICU	LWIR, (1024 × 768)	Piezo-plethysmography ECG	RR	No	RMSE = 0.31–3.27 bpm RMSE = 4.15 bpm
Cho et al. (2017) [55]	23 healthy adults	Lab/Outdoors	LWIR, (160 × 120)	Respiration belt	RR/IBI	No	correlation = 0.9987 RMSE = 0.459 bpm
Cho et al. (2017) [56]	8 healthy adults	Lab	LWIR, (120 × 120)	Instructed protocol	stress level based on RR	No	accuracy = 84.59%/56.52%
Hochhausen et al. (2018) [57]	28 adults	PACU	LWIR, (1024 × 768)	Chest impedance ECG monitor	RR	No	correlation = 0.607–0.849
Chan et al. (2019) [58]	27 adults	ICU	LWIR, (382 × 288)	Chest impedance Manual annotations	RR	No	mean bias = −0.667/−1.000 bpm correlation = 0.796–0.943
Jakkaew et al. (2020) [59]	16 healthy adults	Lab	LWIR, (640 × 480)	Respiration belt	RR	Yes	RMSE = 1.82 ± 0.75 bpm
Jagadev et al. (2020/2022) [60,61]	50 healthy adults	Lab	LWIR, (320 × 240)	Manual annotations	RR	No	[60] precision = 98.76% sensitivity = 99.07% [61] accuracy = 98.83–99.5%
Lorato et al. (2020) [62]	7 premature newborns	NICU	LWIR, (60 × 80)	Chest impedance	RR	No	MAE = 2.07 bpm
Lorato et al. (2021) [63]	9 premature newborns	NICU	LWIR, (60 × 80)	Chest impedance	Apneas	Yes	accuracy = 83.20–94.35%
Kwon et al. (2021) [64]	101 adults	PACU	LWIR, (320 × 240)	Manual annotations Chest impedance	RR	No	correlation = 0.95
Lyra et al. (2021) [65]	26 adults	ICU	LWIR, (382 × 288)	Chest impedance	RR	No	MAE = 2.69 bpm
Takahashi et al. (2021) [66]	7 adults	Lab	LWIR, (320 × 256)	Instructed protocol	RR	No	MAE = 0.66 bpm
Shu et al. (2022) [67]	8 healthy adults	Lab	LWIR, (320 × 240)	PPG	RR	No	error < 2%

alpha—Cronbach’s alpha intraclass correlation; CAND—Absolute normalized difference; ECG—Electrocardiogram; HR—Heart rate; IBI—Interbeat interval; ICU—Intensive care unit; kappa—Chance-corrected agreement; LWIR—Long-wave infrared; MAE—Mean absolute error; MWIR—Mid-wave infrared; OSA—Obstructive sleep apnea; PACU—Postanesthesia; care unit; RMSE—Root mean square error; ROI—Region of interest; RR—Respiration rate; rTV—Tidal volume; NICU—neonatal intensive care unit; PEDS—Pediatric ward; *tp*—Percentage of cycles where error <1 BPM.

In 2015, Pereira et al. started a series of studies with thermal cameras [48,51,53,54]. A new algorithm to detect and track the ROI and estimate the breathing rate was presented. The first study [48] included 11 healthy participants, and a piezo plethysmography (thoracic effort) sensor was used as a reference. These studies laid an important background for breathing signal processing and respiratory disorders detection. In addition, Pereira et al. presented the first study that did not rely on the detection of facial landmarks to measure the respiration signal [54].

Ruminski et al., between 2016 and 2017, developed new ways to measure RR from thermal videos of healthy adults [49,50,52]. The main novelty of Ruminski’s approach consisted of different estimators to extract the RR from the breathing signals. They achieved an MAE between 0.415 and 1.291 bpm in [49,50] and between 0.236 and 0.350 bpm in [52].

In 2017, Cho et al. presented two studies where they used the FLIR ONE thermal camera to measure the respiration rate in highly dynamic scenes [55] and to detect stress situations based on changes in the breathing patterns [56]. These studies presented the results of 23 and 8 healthy participants, respectively. In the first study, the correlation found between the breathing rate acquired through thermal imaging and from the reference respiration belt sensor was 0.99. The second study concluded that there was an accuracy of 84.59% in distinguishing between two levels of stress with thermal imaging.

In 2019, Hochhausen et al. implemented the algorithm developed by Pereira et al. [48] in a real clinical setting [57]. For that, 28 patients were monitored in the post-anesthesia care unit (PACU) with a thermal camera positioned at the foot of the bed. The results showed a Spearman’s rho correlation coefficient of 0.607 on arrival to the PACU and 0.849 upon discharge. Lower respiration rates represented higher correlation values (RR < 12 breaths/min, R = 0.845), whereas higher respiration rates resulted in lower correlation values (RR > 15 breaths/min, R = 0.458), showing the need for improvement of the algorithm.

Chan et al. [58], also in 2019, recruited 27 adults admitted to the intensive care unit (ICU) to monitor their respiration rate with a thermal camera placed at two different distances (0.4–0.6 m and > 1 m). As a reference, chest impedance through ECG electrodes and manual annotations from qualified observers were used. However, these two methods had a limited Pearson correlation coefficient of 0.683. A combination of these two methods was used as the ground truth. The study, which also used Pereira et al.’s algorithm [48], obtained a Person correlation coefficient of 0.960 for the closest distance and 0.508 for the furthest distance. Nevertheless, considering the proximity of the camera to the participants, the first setup is not feasible in a normal clinical setting.

Between 2020 and 2022, Jagadev et al. [60,61] used machine learning approaches to monitor the respiration rate with the FLIR A325 infrared camera placed at a distance of 1 m from the participants. The first algorithm developed reached a precision of 98.76% and a sensitivity of 99.07% while the second study obtained accuracies between 98.8 and 99.5%, bringing a new perspective into the way of filtering the collected signals.

Jakkaew et al. [59] used a portable thermal camera (Seek Thermal Compact PRO for an Apple iPhone) attached to a phone to measure the RR. The camera was placed at 1 m from the side of each of the 16 participants and the reference data were collected from the Go Direct respiratory belt. The study obtained a root mean square error of 1.82 breaths/min and, just like Pereira et al. [54], presented an interesting method to monitor the respiration rate without detecting facial landmarks.

Between 2020 and 2021, Lorato et al. published a series of studies in which they tried to assess respiration rate and to detect apnea events in premature newborns [62,63,68]. A setup with three FLIR Lepton cameras positioned on the side (facing the baby’s face) and at the foot side of the bed was used. Just like Pereira et al. [54] and Jakkaew et al. [59], no facial landmarks were detected. The reference measures were obtained using chest impedance (CI). In [62], the respiration rate was obtained with a mean absolute error of 2.07 breaths/min. In [68], the thermal images were combined with RGB recordings to detect and classify motion and define whether the RR can be extracted. The mean absolute error (MAE) for recording segments with some motion present was 1.97 breaths/min and the overall MAE was 5.36 breaths/min (both from the validation set). It is important to note that the respiration rate for newborns is between 30 to 60 breaths/min, whereas for adults is 12 to 18 breaths/min. In [63], Lorato et al. developed a method to separate the respiration flow from the respiration motion in thermal videos that obtained an accuracy of 84%. In this same study, to prove the relevance of detecting respiration flow pixels, obstructive apneas (OAs) were simulated and then detected. This revealed an improvement in the detection method of OA.

Kwon et al. [64], in 2021, performed a study with a population of 101 adults in the PACU. For each patient, a 2-min video was recorded with a thermal camera and, as references, the RR obtained through chest impedance was used and the number of breaths during one minute was also manually registered. The results showed a correlation between the two reference methods of 0.65 and a correlation between the manual annotations and the remote thermography of 0.95.

Lyra et al. [65] measured the respiration rate of 26 patients in the ICU using a thermal camera mounted to the ceiling of the room. With deep learning algorithms to isolate the chest and the head of the patients, they achieved an MAE of 2.69 bpm.

Also in 2021, Takahashi et al. [66] used the Boson320 camera to measure the respiration rate of seven volunteers. Their method consists of using a deep learning approach to segment the face of the subject and a respiratory likelihood index to find the respiration signal. They achieved an MAE of 0.66 bpm.

In 2022, Shu et al. [67] did a lab study with eight healthy volunteers where thermal videos were recorded while they followed an instructed protocol. In this protocol, the volunteers were asked to simulate three different breathing speeds and to either keep their heads still or to create some head movements. A wearable device was used as a reference. Shu et al. managed to achieve an error lower than 2% on these recordings.

Based on the research of Lorato et al. [62] on respiration extraction, Alves et al. performed some additional research on the number of cameras needed and camera positioning for accurate assessment of respiration rate during sleep [69]. It was concluded that the number of cameras and their placement do not influence the quality of results as long as the subject’s face is visible. This study shows how practical and easy to implement thermal cameras can be to facilitate the patients and the clinical staff.

### 2.2. Defining and Tracking the Region of Interest (ROI)

Defining the region of interest (ROI) is one of the most important steps while extracting the breathing signal from thermal videos. This depends on the camera view or placement used and can also affect the way the signal is processed.

The respiration can be detected in thermal videos in several places and, therefore, the ROI can also have different locations. Most studies focus on the nostrils and surrounding areas, as exhaled air is warmer than inhaled air, creating a detectable thermal contrast. Nevertheless, some studies look for the thermal signature related to breathing movements or even detect respiration flow outside of the subject’s body.

**Figure 3:**
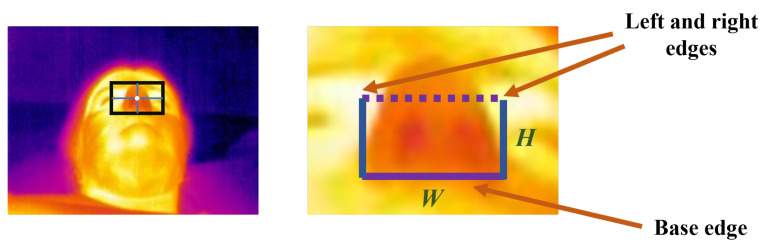
Recreation of a thermal image acquired by Murthy et al. in their 2009 study [40] and respective ROI computed.

In their 2006 study [39], Murthy et al. used a lateral view of the participant and selected the ROI as a square underneath the tip of the nose and between the nostrils and the mouth. The selection of the initial position of the ROI is semi-automatic since the user can adjust or select it. Subsequently, the tracking algorithm follows the ROI as long as the user does not rotate his head or the source of airflow does not change. Later in their work, in 2009 [40], Murthy et al. used a view of the subject’s face at the foot end of the bed. From that view, the ROI is defined as a rectangle around the nostrils. The nostril can be segmented from the rest of the image by detecting the colder boundaries (the cartilage areas) through the application of a Sobel edge detector [70] on the original thermal images. In this study, Murthy used the coalitional tracking system for the facial tissue based on advanced statistics [71], and, for each frame, the ROI was computed. An example of a thermal image acquired during this work with the computed ROI is presented in Figure 3.

**Table 2:** Summary of the techniques used for the region of interest detection and tracking used by the different studies.

	ROI Definition	Body Area	Tracking	Method
Murthy et al. (2006) [39]	Manual	Nostrils/mouth	Yes	ROI adjusted manually; Tracking assumes the relative position towards the tip of the nose
Murthy et al. (2009) [40]	Automatic	Nostrils	Yes	ROI segmentation based on integral projections and an edge detector; Coalitional tracking [71]
Fei and Pavlidis et al. (2010) [41]	Automatic	Nostrils	Yes	ROI detection based on vertical and horizontal gradients; Coalitional tracking [71]
Al-Khalidi et al. (2010) [42]	Automatic	n.a. *	Yes	Two methods for ROI detection based on low pixel intensity; Tracking the circle around the ROI center
Abbas et al. (2011) [43]	Manual	Nostrils	No	-
Lewis et al. (2011) [44]	Manual	Nostrils	Yes	Manual selection of first ROI; PBVD tracking algorithm [72]
Goldman et al. (2012) [45]	Manual	Nostrils, thorax, and abdomen	No	Manual selection of ROIs; Frames differencing
Chauvin et al. (2014) [46]	Manual	Nose/mouth	Yes	TLD algorithm: Tracking based on Lucas–Kanade algorithm [73]; Detector (if needed to reinitialize the tracker); Look at Pose to adjust pan–tilt unit
Pereira et al. (2015/2018) [48,53]	Automatic	Nose	Yes	ROI obtained through a sequence of thresholding, temperature projections, and edge detections; Tracking using the least-squares approach [74]
Ruminski et al. (2016) [49,50]	Manual	Nostrils/nose	No	
Pereira et al. (2017) [51]	Automatic	Nose, mouth and shoulders	Yes	
Ruminski et al. (2017) [52]	Manual	Nostrils/mouth	No	ROI selected should be big enough to account for small movements
Pereira et al. (2018) [54]	Automatic	n.a. *	No	“Black box” approach: a grid is laid over the video and each grid cell is an ROI
Cho et al. (2017) [55,56]	Automatic	Nostrils	Yes	Pre-processing: optimal quantization; Thermal gradient map and gradient through Kalal et al.’s algorithm [75]; Lucas-Kanade’s disparity-based tracker [73]; ROI update
Hochhausen et al. (2018) [57]	Manual	Nose	Yes	Tracking using Mei et al.’s algorithm [74];
Chan et al. (2019) [58]	Manual	Nostrils	Yes	Tracking using Kanade–Lucas–Tomasi tracker [73,76]
Jakkaew et al. (2020) [59]	Automatic	n.a. *	No	Noise removal with a Gaussian filter; ROI considered the square around the highest intensity pixel or ROI is the largest area above a certain threshold
Jagadev et al. (2020) [60]	Manual	Nostrils	Yes	Tracking using the algorithm proposed by Kazemi et al. [77]
Lorato et al. (2020) [62]	Automatic	n.a. *	No	Combination of three features (pseudo-periodicity, RRclusters, and gradient); Core pixel defined as the highest value in the combined matrix; ROI defined as a region with high correlation to the core pixel
Lorato et al. (2021) [63]	Automatic	n.a. *	No	Same method as in [62] with two more features (covariance and flow map) used to separate the motion from flow ROI
Kwon et al. (2021) [64]	Manual	Nose	No	-
Lyra et al. (2021) [65]	Automatic	Head and chest	Yes	Deep learning method: *YOLOv4-Tiny* object detector to extract the ROI continuously [78]
Takahashi et al. (2021) [66]	Automatic	Face	No	Deep learning method: *YOLOv3* to detect the ROI; The ROI is divided into subregions [79]
Jagadev et al. (2022) [61]	Automatic	Nostrils	Yes	Deep learning method (*ResNet50*) for face detection; Tracking using the algorithm proposed by Kazemi et al. [77]
Shu et al. (2022) [67]	Automatic	Nostrils	Yes	Deep learning method: *YOLOv3* to detect and track the ROI

ROI—Region of interest; PBVD—Piecewise Bézier volume deformation model; TLD—Tracking, learning and detection; * non-applicable: no specific body area is detected and tracked.

Fei and Pavlidis [41] used a coalitional tracking algorithm to track facial tissue during recordings by analyzing the spatial distribution of the filter tracker’s clusters. The algorithm follows a tracking ROI that comprises the nostril region where the measures are therefore performed.

Al-Khalidi’s work [42], in 2010, focused on the detection and tracking of the optimal ROI defined as the skin area centered on the tip of the nose. The method detects facial features and uses them to define the ROI. Initially, the images are enhanced with a median filter (size 5) and, subsequently, the face is segmented from the background. The ROI can be computed using one of two methods. The first method entails that the tip of the nose is the lowest intensity pixel in the central region of the face (coldest point in the face), whereas the second method suggests that the tip of the nose is the lowest intensity pixel in between the two regions with the highest intensity pixels, the eye corners. Both methods then construct the ROI as a circle around the tip of the nose. This study considered that the second method had a lower failure rate.

In their work in the neonatal intensive care unit (NICU) [43], Abbas et al. selected the ROI manually as the nostril region taking over the infant’s mouth. There was no automatic ROI definition or tracking involved, therefore this method is only applicable when there is little to no movement of the subject.

Lewis et al. [44] used the Piecewise Bézier Volume Deformation model (PBVD) in their work, which a model that used smooth 3D manipulation to track, through control points, the movement of specific facial features; in this case, the nostrils [72].

Goldman at al. [45] manually defined three ROIs located in the nose area, the thorax, and the abdomen. Consecutive frames were subtracted to remove stationary pixels and enhance the positive and negative variations of the breathing patterns.

Chauvin et al. [46] used a tracking, learning, and detection algorithm (TLD). After a manual selection of the ROI, this algorithm is able to track the ROI and even detect if there is a need for reinitialization. The tracking is based on the Lucas–Kanade algorithm [73] and the learning is done using a semi-supervised approach. Finally, the position of the ROI is used to adjust the camera position in the pan–tilt unit.

In their initial studies, Pereira et al. [48,51,53] used an automatic ROI detection for the first frame that is tracked using the least-squares algorithm developed by Mei et al. [74]. To detect the ROI, the face is initially segmented using a multi-level Otsu threshold [80] where the background noise is removed and the remaining area is the subject’s face. Then, several steps of thresholding, temperature projections, and edge detections are performed to allow the visualization of periorbital regions (warmer regions) to detect the nostrils (in [48,53]) or the mouth and the shoulders (in [51]). To improve the signal-to-noise ratio (SNR), a smaller region inside the ROI was defined named the region of measurement (ROM), which is used to extract the breathing signal. In the study in 2018, Pereira et al. [54] used a “black box” approach where no features were extracted to define the ROI. In this study, a grid was laid over the video and each grid cell was treated as an ROI to extract the respiration signal and RR.

Ruminski et al. defined the ROI manually as the nostrils/nose [49,50] or as the region containing the nose and the mouth [52], ensuring that the ROI is big enough to account for small movements of the subject.

Cho et al. developed a nostril tracking algorithm that initially does an optimal quantification as a pre-processing [55]. This way, there is a conversion between the absolute temperature distributions to color-mapped images. The resulting output is then used to compute the thermal gradient map where feature points can be detected and tracked by Lucas–Kanade’s disparity-based tracker [73] and Kalal et al.’s median flow algorithm [75].

In the studies of Chan et al. [58] and Hochhausen et al. [57], the ROI was manually selected and a tracking algorithm for the ROI was used. Chan and colleagues used the Kanade–Lucas–Tomasi feature tracker [73,76] and Hochhausen implemented the algorithm proposed by Mei et al. [74].

Jagadev et al., in 2020 [60], manually detected the nostrils’ region using the FLIR software [81]. Then, to track the defined ROI over time, they used the algorithm proposed by Kazemi et al. [77] that estimates the facial landmarks’ location using an optimization of the sum of square error loss. The facial landmarks correspond to a set of points surrounding the chin area, mouth, nose, eyes, and eyebrows. In 2022 [61], the team developed a deep learning model to automate the face and nostrils detection—the Residual network 50 + Facial landmark detection’ (*ResNet50+FLD*) model.

Jakkaew and colleagues did a pre-processing of the images by applying a Gaussian filter in order to remove noises from the input [59]. Then, two different methods were tested to compute the ROI. The first method considers the ROI as the square around the highest intensity value, whereas the second method defines ROI as the largest area above a certain threshold. The two methods were not compared but combined in the next step to extract the respiration signal.

**Figure 4:**
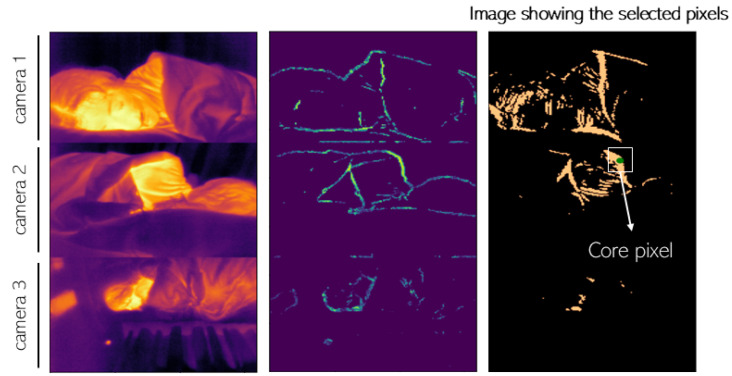
(**left**) Example of an acquired thermal image (from three cameras); (**middle**) matrix combining all the extracted features; (**right**) ROI selected pixels [62].

Following the same idea as Pereira et al.’s study [54], Lorato et al. presented an approach where the ROI detection did not rely on facial landmarks [62,68]. These approaches become relevant since they are focused on the presence of a breathing signal and not on facial features that might require specific camera angles or viewing perspectives. To do that, Lorato et al. combined three different features to find a core pixel that is correlated to the rest of the ROI. The features used are the pseudo-periodicity, which can be defined as the predominant frequency of each pixel; the respiration rate clusters, which consist of a measure of similarity of one pixel with its neighboring pixels; and the thermal gradient, which is a measure of thermal contrast. The normalized matrices for each feature are combined into one matrix where the highest intensity pixel is considered the core pixel. The core pixel becomes the pixel that is more likely to have a high-quality respiration signal. The ROI contains all the pixels whose Pearson correlation value with the core pixel is higher than 0.7. Figure 4 contains an example of the acquired thermal images, the matrix combining all the features, and the ROI selected pixels. In their later study, in [63], Lorato et al. also introduced two more features that were subsequently used to distinguish respiration flow (RF) from respiration motion (RM). These features are the covariance map and the flow map. The flow map is a combination of all the features that is used as an input to a Gabor bank of filters, empirically defined, in order to enhance pixels where flow is most likely to be present.

Kwon et al. [64] manually defined the ROI as a region around the nose, and, considering that each recording was 2 min, no tracking of the ROI was performed. Nevertheless, data from six patients were priorly excluded due to excessive movement.

Also in 2021, Lyra et al. [65] implemented the *YOLOv4-Tiny* deep learning model [78] to locate the head and chest of the patients. This model was trained using manually labeled frames and was also able to identify any clinical staff within the field of view, enabling real-time monitoring.

Similarly to Lyra et al., Takahashi et al. [66] used the *YOLOv3* deep learning model [79] to detect the face of the subject. The face region is then divided into several subregions where a respiratory likelihood index is computed. This value, ranging between 0 and 1, is related to the most respiratory-related areas, usually located around the nose and the mouth.

Also using the *YOLOv3* deep learning model [79], Shu et al. [67] were able to create a system that detects and tracks the nostrils. This model achieved accuracies of 97.9% with both static and moving conditions.

Some other studies investigated further the choice of ROI and how it can be affected, for instance, by medical devices. Huang et al. [82], in 2021, used a deep alignment network to extract the mouth and nose area. They used this information to classify whether a person was breathing through the nose or the mouth. In 2022, Koroteeva et al. [83] analyzed the effect of the use of a face mask on the distribution of heat in the face and on the dispersion of airflow. Telson et al. [84], in 2024, compared three different ROIs: a line, a rectangle, and an ellipse, all in the mouth and nostrils area, simultaneously. They concluded that both the rectangle and the ellipse ROI shapes significantly detect the temperature changes between inhalation and exhalation.

The selection of the ROI is a fundamental step in monitoring respiration with thermal cameras. Although most of the methods mentioned focus on the nose and nostrils area, the ROI can also be located in other parts of the body or even, for instance, on the pillow due to the heating waves of the breathing airflow. The robustness of the ROI detection will depend on how controlled the conditions for measurement should be. Having a method that does not require the detection of facial landmarks and is prepared to find the ROI in several areas will ultimately be less prone to errors. Finally, a method that can distinguish ROIs associated with respiration motion from respiration flow will provide more information and, therefore, can be used in more applications.

### 2.3. Breathing Signal Extraction and Respiration Rate Estimation

Following the definition of the ROI is the extraction of the breathing signal that can be used to estimate the respiration rate (RR). In this step, the respiration signal can also be distinguished between respiration flow and respiration motion.

After characterizing the ROI, Murthy et al. [39], in 2006, performed a 3-step operation in the acquired videos to enhance the visualization of the inspiration and expiration phases. The core step of this operation consisted of generating the differential infrared thermography where a breath mask was created from the pixels whose value increased above a certain threshold. These pixels should contain the breathing information. The temperature together with the ROI size information is used to train the model that labels the pixels as expiratory or non-expiratory. This labeling occurs for different frames over time, and the duration of the expiratory and non-expiratory cycles gives an estimation of the breathing rate of the subject. In their later work, in 2009, [40], Murthy et al. collected the breathing waveform by applying a continuous wavelet transform (CWT) in the normalized thermal signal of the ROI that is defined as the pixel mean intensity value over time. The breathing signal is then considered the strongest component of the CWT.

**Table 3:** Summary of the techniques used for breathing signal extraction and RR estimation used by the different studies.

	Breathing Signal Extraction and RR Estimation Methods
Murthy et al. (2006) [39]	- Breathing waveform as the number of pixels and their temperature
Murthy et al. (2009) [40]	- Respiration signal as the averaged intensity of ROI - Wavelet analysis CWT
Fei and Pavlidis et al. (2010) [41]	- Respiration signal as the averaged intensity of ROI - Wavelet analysis CWT
Al-Khalidi et al. (2010) [42]	- Respiration signal as the averaged intensity of ROI
Abbas et al. (2011) [43]	- Respiration signal as the averaged intensity of ROI - Wavelet analysis CWT (Debauchies wavelet)
Lewis et al. (2011) [44]	- Thermal signal as the averaged intensity of each nostril - Respiration rate measured through the spectral density distribution - Tidal volume measured through thermal signal integration - Dynamic filtering
Goldman et al. (2012) [45]	- Respiration signal as the difference between positive and negative areas - Phase correction and filtering (Chebyshev) - Fourier transform to obtain the RR
Chauvin et al. (2014) [46]	- Gradient to mask the ROI - Breathing waveform as the average intensity within the mask - Hanning window and Fourier transform to obtain the RR
Pereira et al. (2015/2018) [48,53] Chan et al. (2019) [58] Hochhausen et al. (2018) [57] Kwon et al. (2021) [64]	- Respiration signal as the average intensity of the ROM - Filtering: Butterworth - IBI computed with the Brüser et al. algorithm [85]: three estimators combined with a Bayesian function
Ruminski et al. (2016) [49,50]	- Respiration signal as the averaged intensity of the ROI - Signal normalized and filtered (moving average and Butterworth filters) - RR extracted using four different estimators
Pereira et al. (2017) [51]	- Respiration signal of the nose and mouth ROIs as the average intensity - Respiration signal of the shoulders as the vertical movement - Fourier transform to extract RR - SQI computation based on four features of the power spectrum - Fusion algorithm to combine all regions
Ruminski et al. (2017) [52]	- Respiration signal computed using a skewness operator - Filtering: Butterworth - RR extracted using three different estimators
Cho et al. (2017) [55]	- Respiration signal computed through a thermal voxel-based method - RR determined through short-time power spectral density: Fourier transform of the short-time autocorrelation function
Cho et al. (2017) [56]	- Computation of the 2D spectrogram - Data augmentation - CNN to classify different stress levels
Pereira et al. (2018) [54]	-For each grid cell: - Hamming window, Fourier transform, normalization, and filtering - SQI computation based on four features of the power spectrum -Selection of cells with SQI > 0.75 -RR defined using three different fusion techniques
Jakkaew et al. (2020) [59]	- Respiration signal as the averaged intensity of the ROI - Filtering: Butterworth, Savitzky–Golay, and moving average - RR computed through the number of peaks in the signal
Jagadev et al. (2020) [60]	- Respiration signal as the averaged intensity of the ROI - Testing and comparing different filters - Breath detection algorithm to extract the RR
Lorato et al. (2020/2021) [62,63]	- Respiration signal as the averaged intensity of the ROI - Filtering: Butterworth - RR as the predominant frequency
Lyra et al. (2021) [65]	- Optical flow algorithm [86] to detect pixel intensity changes - RR as the frequency of the changes
Takahashi et al. (2021) [66]	- For each subregion: -Frequency analysis: PSD -Respiratory likelihood index as a weighted score of the PSD - RR as the frequency with the highest index
Jagadev et al. (2022) [61]	- Machine learning algorithm (*BSCA*) to automatically obtain the RR
Shu et al. (2022) [67]	- Respiration signal as the average intensity of the ROI - Filtering: Butterworth - RR as the predominant frequency

BSCA—Breathing signal characterization algorithm; RR—Respiration rate; ROI—Region of interest; CWT—Continuous wavelet transform; ROM—Region of measurement; IBI—Inter-breath interval; SQI—Signal quality index; CNN—Convolutional neural network; IIR—Infinite impulse response; PSD—Power spectral density.

Fei and Pavlidis [41] also used a wavelet analysis to extract the breathing rate from the ROI of the thermal videos. For that, the signals were resampled to 10 Hz and normalized before applying a CWT. The center frequency of the mother wavelet, adjusted for down sampling, is the final RR. Abbas et al. [43] also applied a CWT to the average intensity of the pixels in the ROI. They used the Debauchies (Db) transformation to decompose the signal since it provides better results for biomedical signals [87].

Using the manually selected ROI, Al-Khalidi [42] extracted the breathing signal as the average pixel value of that region, filtered using a bandpass filter with cutoff frequencies between 0.05 and 1 Hz (3 to 60 breaths/min).

In their study, Lewis et al. [44] considered the breathing signal as the average of two mean temperatures of each nostril. The breathing signal was then detrended and zero-padded before applying the FFT. The spectral density distribution obtained was used to determine which was the predominant frequency between 3 and 65 breaths/min, which was considered to be the RR. Lewis also integrated the thermal time series, which generated a linear time series related to the tidal volume.

Goldman et al. [45] obtained the breathing signal of each ROI by computing the difference between the positive and negative areas defined in the previous step. The signals were then phase corrected and filtered with a low pass Chebyshev filter. A Fourier transform was applied to extract the RR as the spectral maximum peak.

Chauvin et al. [46], in 2014, used the Sobel operator to obtain an approximation of a gradient used to mask the ROI. The breathing signal is then considered as the average pixel intensity within the mask region. They apply a Hann window and a Fourier transform to the signal and define the RR as the predominant frequency between 0.2 and 0.5 Hz (12 to 30 breaths/min).

Pereira et al., in 2015 [48,53], defined the breathing signal as the mean temperature of the ROI over time. To extract the RR from that signal, C. Pereira filtered the signal with a Butterworth band-pass filter with cutoff frequencies of 0.1 and 0.85 Hz (6 and 51 breaths/min) and then applied the algorithm of Brüser et al. [85,88]. This algorithm uses three estimators that are then combined with a Bayesian fusion method. The three estimators are the adaptive window autocorrelation that computes the correlation for different frames in a window of frames, the adaptive window average magnitude difference function that finds the absolute difference between samples in an adaptive window, and the maximum amplitude pairs that can be defined as an indirect peak finder. The Bayesian fusion method that combines these three estimators returns a value for the inter-beat interval. Chan et al. [58], Hochhausen et al. [57], and Kwon et al. [64] used the same method in their work.

In 2018, Pereira et al. [54] estimated the RR using four ROIs: the nose area, the mouth area, and the two shoulders. The breathing waveform of the nose and mouth ROIs was defined as the mean pixel intensity, and the breathing waveform of the shoulder ROIs was considered as their vertical movement. The signals were processed and the RR was computed through the Fourier transform. For each ROI signal, the signal quality index (SQI) was determined based on four features of the normalized power spectrum. The signals from all ROIs were combined using three different approaches: the median of all signals, the best SQI, and the Bayesian fusion.

In a later work [54], Pereira and colleagues used a different approach to extract the RR from thermal videos. For that, they placed a grid over each frame and treated each grid cell separately. For each grid cell, they applied a Hamming window and a Fourier transform and then normalized the signal to its maximum value. Subsequently, they computed the breathing signal by filtering the signal between 0.1 and 3 Hz (6 to 180 breaths/min) and the SQI using the same method as their previous work. The grid cells with a SQI higher than 0.75 were selected as the final ROI and the RR was computed using three different fusion techniques, similar to the ones previously used.

Ruminski et al., in [49], extracted the respiration waveform as the average intensity level of the manually defined ROI. The signal was then normalized, filtered with a moving average operation (window size of five frames), and filtered with a Butterworth high pass filter with a cutoff frequency of 0.1 Hz. To find the respiration rate, Ruminski used four different estimators: eRR_sp considered the highest peak in the frequency spectrum, eRR_ac based on the periodicity of the signal peaks, eRR_zc based on the number of zero crossings, and eRR_pk that uses the information of the number of peaks. It was concluded that the estimator eRR_ac was the one that delivered the best results when compared to the reference method. Nevertheless, these estimators were not combined at any point during the study.

In their later study, Ruminski et al. [52] introduced the skewness operator to extract the respiration waveform. Skewness measures the symmetry or lack of symmetry of data and the inspiration patterns create changes in the skewness parameter. The RR was computed using three parameters similar to the ones described in their previous study. Ruminski et al. concluded that the skewness operator largely improved their last results.

Cho et al., in [55], used a thermal voxel-based method to define the respiration signal by mapping the thermal units into three-dimensional space and computing the volume changes. This enhances the quality of the signal. The RR was determined using a short-time power spectral density. That consists in applying the Fourier transform to the short-time autocorrelation function using a Gaussian window. The obtained signal is filtered between 0.1 and 0.85 Hz (6 to 51 breaths/min) and the RR is defined as the value that maximizes the power spectral density.

Also in 2017, Cho et al. [56] used a convolutional neural network (CNN) to classify different stress levels. As an input, they used two-dimensional spectrograms that were computed by stacking the power spectral density vector (PSD) over time. The data were augmented while preserving each label. The final CNN architecture consisted of two convolutional layers, two pooling layers, and one fully connected layer.

In Jagadev’s work [60], the breathing waveform was collected from the ROI and filtered using different types of infinite impulse response (IIR) filters. To understand which filter presented the best results, several parameters were computed, such as SNR, mean square error, magnitude response, and group delay. Jagadev concluded that the Butterworth filter showed the best performance. Subsequently, a breath detection algorithm was applied to the filtered signal that iterates several steps to improve the detection of breathing cycles. This algorithm takes into consideration the number of peaks and valleys of the signal as well as the cycle duration of waveforms with an SNR above a certain threshold. In their follow-up work [61], the team used a machine learning approach to automatically detect the RR—the “*breathing signal characterization algorithm (BSCA)*”. In addition, two classifiers were tested: the *decision tree* and the *support vector machine*; the last one had the best results.

Jakkaew et al. [59] also applied several filtering steps to the breathing signal collected as the average pixel intensity within the ROI. These steps consisted of using a Butterworth filter (0.05 to 1.5 Hz), followed by a Savitzky–Golay filter, and then a moving average filter. The RR is then settled as the number of peaks in the signal per time unit.

In I. Lorato’s work [62,63], the breathing signal was also computed as the average pixel intensity within the ROI. The signal was filtered with a Butterworth filter and the RR was considered the predominant frequency in the frequency spectrum of the signal.

Lyra et al. implemented an optical flow algorithm [86], a computer vision technique, that detects motion by tracking changes in pixel intensities. It measures the displacement of pixels over time within the chest region that is associated with breathing movements. The algorithm is able to compute the frequency of these motion patterns, i.e., the RR in real time.

In their study, Takahashi et al. [66] defined the respiratory likelihood index for each subregion of the face as a measure of the most respiratory-related areas. For that, they compute the temperature signal for each subregion as the average temperature over time. That signal goes through a frequency analysis. The respiratory likelihood index is a weighted score of the power spectral density at respiratory-related frequencies. The frequency with the highest index is, therefore, the RR.

In 2024, Shu et al. [67] also defined the breathing waveform as the average pixel intensity within the ROI. They compared two low-pass filter options: the Chebyshev and the Butterworth, ending up opting for the last one. The RR value was computed as the dominant frequency in the obtained signal.

## 3. Applications

Thermal cameras are suitable for continuous respiration monitoring due to their non-contact nature. Their applications can go from clinical use, such as monitoring patients in ICUs or neonatal units, to home care settings for managing chronic conditions like COPD. Besides giving information on respiration rate, thermal cameras can be used to evaluate changes in respiration flow or cessation of breathing. Apnea is defined as the complete cessation of airflow for at least 10 s, whereas hypopnea is a partial reduction in airflow or breathing with a significant decrease in oxygen levels. Considering this, detecting or identifying apnea or cessation of breathing with thermographic cameras becomes an area of interest. In addition, since thermal cameras do not require an illumination source, their application can also be extended to sleep monitoring and apnea detection. Even though this is still an area under development, several studies of the ones previously mentioned worked towards these utilizations.

### Apnea Detection

In the study of Pereira and colleagues [48], participants were asked to simulate different breathing patterns such as eupnea (normal spontaneous breathing), tachypnea, apnea, and deep breathing. The breathing frequency obtained with thermal imaging was compared with the piezoplethysmography signal through a correlation. The results revealed an average correlation of 0.974 between the two methods. In addition, Pereira concluded that the disparity between the two methods occurred mainly during the transitions and the error outside this area was very small. Nevertheless, there was no automatic classification algorithm implemented.

In 2016, Ruminski [49] used four different estimators to compute the respiration rate. They concluded that, if the values of the estimators are significantly different, there is a high chance that there is a cessation of respiration airflow. In these cases, an apnea detector should be used, but this was not implemented in their study.

P. Jagadev [60] also implemented a classifier in order to distinguish four different classes: normal breathing, abnormal breathing, tachypnea, and bradypnea. It used a 10-fold cross-validation k-nearest neighbor (k-NN) classifier for multi-class classification. The data showed it to be well separated with minimal overlap and, therefore, proved this approach to be accurate.

The most recent study that delivered some insights into apnea classification was the work by Lorato et al. in 2021 [63] that used recordings from infants in the neonatal medium care unit. Lorato classified the pixels in a thermal video into RF (respiration flow), RM (respiration motion), or MR (mixed respiratory) pixels. This classification distinguishes between obstructive apnea (OA) and central apnea (CA). In CA, there is little to no RM or RF detected and it can be comparable to a breath hold event. In OA, there can be an increase in the respiratory effort—RM [89,90]—but there is a reduction in the RF and, in the case of infants, even a total absence. To distinguish the different types of pixels, Lorato considered that the RM occurs in only one direction (up and down movement of the chest/abdominal area), whereas the RF creates a thermal gradient that spreads around in every direction. Since no apnea events from their dataset were detected, OA was simulated. Lorato et. al replaced the manually annotated RF pixels with noise pixels. Using the last two features mentioned in Section 2.2, the automatic identification of RF pixels and consequently definition of the RF signal was possible. To detect OA, I. Lorato applied a cessation of breath (COB) detection algorithm [91] to the RF signal that consists of the comparison between short-term standard deviation and long-term standard deviations. The final algorithm was able to detect OA with 94.35% accuracy.

## 4. Limitations and Challenges

The adoption of thermal cameras for continuous respiration monitoring presents advantages; however, several factors, including the placement of cameras and the movement of the participants, can influence their accuracy and reliability in this application. Nevertheless, Alves et al. [69] concluded, in a lab study, that changes in camera placement, subject’s static position, and the use of a nasal cannula do not affect the respiration signal quality. The use of thermal cameras usually implies a trade-off between cost and sensitivity to small temperature changes. The cost of high-quality/high-resolution cameras may limit their widespread accessibility. However, using low-cost/low-resolution cameras poses challenges regarding how accurately subtle respiratory movements can be captured, particularly when monitoring from a distance. In addition, environmental conditions, such as external heat sources (e.g., radiator or heated blankets), fluctuations in room temperature, or adjacent medical devices may interfere with the camera’s ability to reliably detect the respiration signal. The accuracy of thermal measurements is also susceptible to the movement of the monitored subject. Movements might be erroneously interpreted as respiratory changes. Furthermore, the positioning of the person in relation to the camera can influence the quality of data, especially if the person is not facing the camera directly. Furthermore, the standardization of the procedure to acquire and process thermal videos for respiration monitoring represents a challenge and entails complex algorithms and signal-processing techniques. In light of these limitations, ongoing research and technological advancements are essential to address these challenges and to further develop its applications.

## 5. Conclusions

The use of thermal cameras to monitor respiration rate and detect apnea cases is being proven to be of great pertinence. This technique detects temperature changes associated with respiration airflow and respiration motion. This can bring significant improvements to the patient’s well-being and to the clinicians’ work and diagnosis methods considering its non-contact nature. Nevertheless, there is still a need to adapt the technologies and methods that have been studied in order to make them implementable in a clinical setting for standard use. These include improving the accuracy and sensitivity of thermal imaging systems, minimizing the impact of environmental factors on measurements, and developing robust algorithms for real-time analysis. Additionally, further studies are needed to validate the technology across larger populations and different clinical conditions. The measurement of the respiration rate should be comparable to the gold standard and deliver values up to the medical requirements. The detection of apnea and the distinction between OA and CA should also match the ground truth given by specialists with adequate accuracy.

Considering all of this, the use of thermal cameras for respiratory monitoring could improve patient care by offering a convenient non-contact alternative to traditional methods. Nevertheless, further research and development in this field is essential to mature this technology for clinical use.

## Data Availability

Data are contained within the article.
